# N-Acetylcysteine, N-Acetylcysteine Amide, and Thioredoxin Mimetic Peptides Regenerate Mercaptoalbumin and Exhibit Antioxidant Activity

**DOI:** 10.3390/antiox13030351

**Published:** 2024-03-15

**Authors:** Sonia Eligini, Marco Munno, Gloria Modafferi, Daphne Atlas, Cristina Banfi

**Affiliations:** 1 Centro Cardiologico Monzino IRCCS, Unit of Functional Proteomics, Metabolomics, and Network Analysis, 20138 Milan, Italy; sonia.eligini@cardiologicomonzino.it (S.E.); marco.munno@cardiologicomonzino.it (M.M.); gloria.modafferi@cardiologicomonzino.it (G.M.); 2 Department of Biological Chemistry, Institute of Life Sciences, The Hebrew University of Jerusalem, Jerusalem 91904, Israel; daphne.atlas@mail.huji.ac.il

**Keywords:** N-acetylcysteine, N-acetylcysteine amide, thioredoxin-mimetic compounds, albumin, antioxidant activity

## Abstract

Albumin (HSA) is the most abundant circulating protein and plays a pivotal role in maintaining the redox state of the plasma. Three HSA proteoforms have been identified based on the redox state of cysteine 34. These proteoforms comprise of the reduced state (HSA-SH) referred to as mercaptoalbumin, non-mercaptoalbumin-1, containing a disulfide with small thiols such as cysteine (HSA-Cys), and non-mercaptoalbumin-2, representing the higher oxidized proteoform. Several clinical studies have shown a relationship between an individual’s serum HSA redox status and the severity of diseases such as heart failure, diabetes mellitus, and liver disease. Furthermore, when HSA undergoes oxidation, it can worsen certain health conditions and contribute to their advancement. This study aimed to evaluate the ability of the redox compounds AD4/NACA and the thioredoxin mimetic (TXM) peptides TXM-CB3, TXM-CB13, and TXM-CB30 to regenerate HSA-SH and to enhance its redox activity. The HSA proteoforms were quantified by LC-MS, and the antioxidant activity was determined using dichlorofluorescin. Each of the compounds exhibited a significant increase in HSA-SH and a reduction in HSA-Cys levels. The increase in HSA-SH was associated with a recovery of its antioxidant activity. In this work, we unveil a novel mechanistic facet of the antioxidant activity of AD4/NACA and TXM peptides. These results suggest an additional therapeutic approach for addressing oxidative stress-related conditions.

## 1. Introduction

Albumin (HSA) is the most abundant circulating protein in humans and constitutes about 50% of the plasma protein in normal subjects. It is involved in several physiological functions, including the modulation of the acid–base balance, the liquid exchange between the intravascular and interstitial compartments, maintaining vascular endothelium integrity, and the binding of endogenous and exogenous compounds. HSA displays various beneficial properties such as anti-inflammatory, anticoagulant, antiaggregating, and antioxidant activity [1,2,3,4].

In this scenario, HSA provides an important antioxidant effect in the plasma, which is continuously exposed to oxidative stress that is induced by reactive oxygen and nitrogen species, resulting in the formation of several oxidation products [5,6,7].

HSA has 35 cysteine (Cys) residues, of which 34 form intramolecular disulfide bonds, while Cys34 is free and redox-active [8,9]. According to the redox status of Cys34, three proteoforms of HSA have been identified: mercaptoalbumin, the reduced proteoform (HSA-SH) with a free thiol group; non-mercaptoalbumin-1, a mixed disulfide with small thiol compounds such as Cys, homocysteine, cysteinylglycine, or glutathione; and non-mercaptoalbumin-2, the higher oxidized proteoform with Cys34 as sulphinic or sulphonic acid [10,11,12]. The reduced and oxidized proteoforms of HSA exhibit distinct physical, chemical, and biological characteristics. While HSA has several residues that are susceptible to oxidation, including tryptophan, tyrosine, and methionine, it is the sulfhydryl group of Cys34 that is essential for regulating the redox function of different proteoforms [13,14]. In vivo, there are various mechanisms that can cause oxidation of HSA, leading to different forms of oxidized HSA.

In healthy young populations, HSA-SH accounts for 70–80%, non-mercaptoalbumin-1 for 20–30%, and non-mercaptoalbumin-2 for 2–5% of the total HSA [15]. Increased levels of oxidized HSA have been detected in several pathological conditions such as heart failure, diabetes mellitus, liver disease, and renal failure [16,17,18,19,20,21,22,23,24,25]. Furthermore, recent clinical studies have demonstrated a relationship between the serum HSA redox status and the severity of these diseases [19,20,21,26,27,28,29,30,31,32]. In addition, in vivo and ex vivo studies have shown that oxidized HSA per se can aggravate several pathological conditions and contribute to their progression [13,17,22,33,34,35,36].

In 2004, Harada et al. demonstrated that N-acetylcysteine (NAC) can rapidly reduce the disulfide bond of HSA-Cys, dissociating Cys from HSA [37]. Recently, we have shown that NAC, through the breaking of the thiol–disulfide bond, regenerates the native form of HSA, with a complete recovery of its antioxidant and antiplatelet properties [38,39,40]. Although NAC is generally safe and well tolerated, its bioavailability is low, typically ranging from 5 to 10% [41,42]. Indeed, at a physiological pH, NAC loses a proton from the carboxyl group, acquiring a negative charge that hinders its passage across the membrane [43]. In this regard, a series of low-molecular, brain-targeted, non-charged, lipophilic thiol compounds with improved membrane permeability and bioavailability have been developed (US Patent No.5874468) [43]. The amide form of NAC, termed AD4/NACA, has been extensively studied. Compared to NAC, it displays higher efficacy as an antioxidant, higher bioavailability, and also crosses the blood–brain barrier, indicating its potential for therapeutic interventions within the central nervous system [43,44]. Additionally, AD4/NACA has demonstrated improved redox activity, anti-inflammatory, and anti-apoptotic properties compared to its parent compound [43,45,46,47,48].

The thioredoxin reductase/thioredoxin (TrxR/Trx) system is one of the major intracellular systems among multiple mechanisms that are involved in maintaining the homeostatic redox state of the cell. This system consisting of Trx, TrxR, and NADPH plays a pivotal role in preserving a reduced intracellular environment through the thiol–disulfide exchange reaction [49]. Cytosolic Trx (Trx1) contains within the active site two redox-active Cys residues (Cys32 and Cys35), which are responsible for maintaining the thiols of the proteins in a reduced form while being oxidized themselves [50].

In addition, it has been shown that thioredoxins have a protective role in several cardiovascular diseases, as such the TrxR/Trx system might be a target for development new clinical therapies in the treatment of cardiovascular pathologies [51,52,53]. Indeed, Trx1 shows both antioxidant and anti-inflammatory activities [54]. It can also modify the gene expression [55], leading to a reduction in ischemia/reperfusion injury [56], myocardial infarction [57,58], and cardiac hypertrophy [59]. Trx2, the mitochondrial isoform, contributes to maintaining the cardiac function, reducing the radical oxygen species generation [60,61] and reducing the vascular dysfunction and hypertension that are caused by angiotensin II [62,63].

A family of small-thiol peptides was developed based on the rational design of the active site of Trx1 and Trx2, the -CxxC motif, along with the -CxC- catalytic motif of protein disulfide isomerase (PDI) [64]. These Trx mimetic (TXM) peptides consist of three or four amino acids, bearing two cysteine residues flanking either two amino acids (CxxC motif) or a single amino acid (CxC motif), and are blocked on both the N- and C-termini.

In this study, we assessed the capability of AD4/NACA and the TXM peptides, TXM-CB3, TXM-CB13, and TXM-CB30, to facilitate the regeneration of mercaptoalbumin and enhance its antioxidant activity.

## 2. Materials and Methods

### 2.1. Blood Collection

This study was performed according to the Declaration of Helsinki and was approved by the local institutional Ethics Committee. Plasma was obtained from three healthy subjects enrolled at Centro Cardiologico Monzino. All the participants provided written informed consent at the time of enrollment, and none of the participants had taken any drugs in the previous 10 days. Venous blood was drawn from the antecubital vein and collected in Vacutainer^®^ tubes containing 0.129 mM sodium citrate, and plasma was obtained after centrifugation at 1000× *g* for 15 min.

### 2.2. Detection of Albumin Proteoforms by LC–Mass Spectrometry

**Figure 1:**
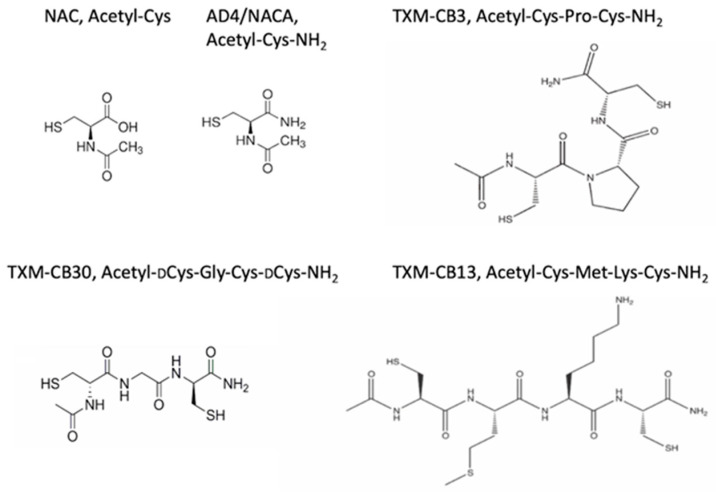
Chemical structure of NAC, AD4/NACA, and the thioredoxin mimetic (TXM) peptides, TXM-CB3, TXM-C30, and TXM-CB13.

NAC-amide (AD4/NACA), TXM-CB3 (Ac-Cys-Pro-Cys-amide), TXM-CB13 (Ac-Cys-Met-Lys-Cys-amide), and TXM-CB30 (Ac-DCys-Gly-DCys-amide) (Figure 1) of >98% purity were synthesized by Novetide Ltd., Haifa, Israel.

Human HSA solution (Albumina Grifols^®^, 200 g/L) or plasma obtained from healthy subjects were incubated at 37 °C with stirring (300 rpm) with NAC, NAC-amide (AD4/NACA), or the TXM peptides (TXM-CB3, TXM-CB13, TXM-CB30). The relative abundance of HSA proteoforms was detected by infusing the sample into a Xevo TQ-S micro triple-quadrupole mass spectrometer coupled with the ACQUITY UPLC^®^ M-Class system (Waters Corporation, Milford, CT, USA), as previously described [21]. Briefly, HSA samples were diluted 4000-fold in a solution containing 30% acetonitrile and 0.1% formic acid and then centrifuged for 10 min at 14,000× *g* at 4 °C. The sample was injected in full loop mode using 2 µL, utilizing an ACQUITY UPLC Protein BEH C4 Column, 300 Å, 1.7 µm, 1 mm × 50 mm, 1/pK (Waters Corporation, Milford, MA, USA) column. The flow rate was set to 5 µL/min, while the column temperature was held constant at 40 °C. A gradient of solvent A, a solution of 99.9% LC-MS-grade water with 0.1% formic acid and solvent B, a solution of 99.9% LC-MS-grade acetonitrile with 0.1% formic acid, was applied as reported in Table 1.

Spectra were acquired for 4 min, using the following parameters: ESI positive mode, mass range between 1100 and 1350 *m*/*z*, MS scan in multi-channel acquisition with 1 s scan time, capillary voltage at 3 kV, cone voltage at 90 V, desolvation temperature at 350 °C, and source temperature at 150 °C. Data processing for deconvolution was performed utilizing the MaxEnt 1TM function on the MassLynx software V4 (Waters Corporation, Milford, CT, USA) with the following parameters: mass range was between 40,000 and 80,000 Da; damage model based on uniform Gaussian 1.1 Da width at half height; minimum intensity ratios for adjacent peaks, left 33% and right 33%; iterated to convergence. Intensities of HSA-SH and HSA-Cys (+120 ± 2 Da) were used to calculate their respective relative abundances, as previously described [21]. The mass of HSA was calculated by a deconvolution algorithm (MaxEnt1), which calculates the centroid of the mass distribution instead of the monoisotopic mass of the protein. For all the tested samples, a coefficient of variation of 7.0 ± 1.1% (mean ± SEM) was calculated for the percentages of HSA-Cys.

### 2.3. Quantification of Free Sulfhydryl Groups

After incubating the HSA solution (200 g/L) or plasma obtained from healthy subjects with NAC, AD4/NACA, or the TXM peptides (TXM-CB3, TXM-CB13, TXM-CB30), free sulfhydryl groups were detected using Ellman’s method, which was modified by Riddles et al. [65,66]. Briefly, 50 µL of Ellman’s reagent (0.4 mg 5,5′-dithio-bis-(2-nitrobenzoic acid)/mL reaction buffer (0.1 M sodium phosphate, pH 8.0 containing 1 mM EDTA)) and 215 µL of reaction buffer were added to 10 µL of HSA solution or plasma. Samples were incubated at room temperature for 15 min. Ellman’s reagent reacts with free sulfhydryl groups to yield a mixed disulfide and highly chromogenic 2-nitro-5-thiobenzoic acid, which was quantified by the absorbance at 412 nm using the molar extinction coefficient 14,150 M^−1^cm^−1^.

### 2.4. Measurement of Antioxidant Activity

The antioxidant activity was assessed in the HSA solution (200 g/L) after incubation with the different compounds. The assay was performed using the 2′,7′-dichlorodihydrofluorescin diacetate (DCFH-DA; Sigma Aldrich S.r.l., Milan, Italy) as fluorescent probe and 2,2′-Azobis (2-amidinopropane) dihydrochloride (AAPH, Sigma Aldrich S.r.l., Milan, Italy) as the radical generator [67]. DCFH was obtained from DCFH-DA by basic hydrolysis: 500 µL of 1 mM DCFH-DA was treated with 2 mL 0.01 M NaOH at 4° C while protected from the light. After 20 min, the mixture was neutralized with 2 mL 0.01 M HCl. Next, 20 µL of the sample was loaded in triplicate in a 96-well black plate (Corning Incorporated Costar, Euroclone S.p.A., Milan, Italy) and diluted with 50 µL phosphate-buffered saline. Finally, 20 µL AAPH (0.1 mM final concentration) and 10 µL DCFH (10 µM final concentration) were added. Oxidation of DCFH to 2′,7′-dichlorofluorescin (DCF) was monitored at 37 °C, setting the excitation at λ 485 nm and emission at λ 535 nm.

### 2.5. Statistical Analysis

Data are expressed as mean ± SD. Differences between the groups were assessed by Student’s *t*-test for single comparison or by one-way analysis of variance (ANOVA) for multiple comparisons and Dunnett’s post hoc test, as indicated. Pearson correlation analysis was performed to test the relationship between HSA-SH and antioxidant activity, detected as units of DCF generated from DCFH oxidation. All tests were 2-sided. A *p* ≤ 0.05 was considered statistically significant.

## 3. Results

### 3.1. Dose-Dependent Increase in HSA-SH and Decrease in HSA-Cys Mediated by N-Acetylcysteine (NAC), N-Acetylcysteine Amide (AD4/NACA), and the Thioredoxin Mimetic (TXM) Peptides, TXM-CB3, TXM-CB13, and TXM-CB30

**Figure 2:**
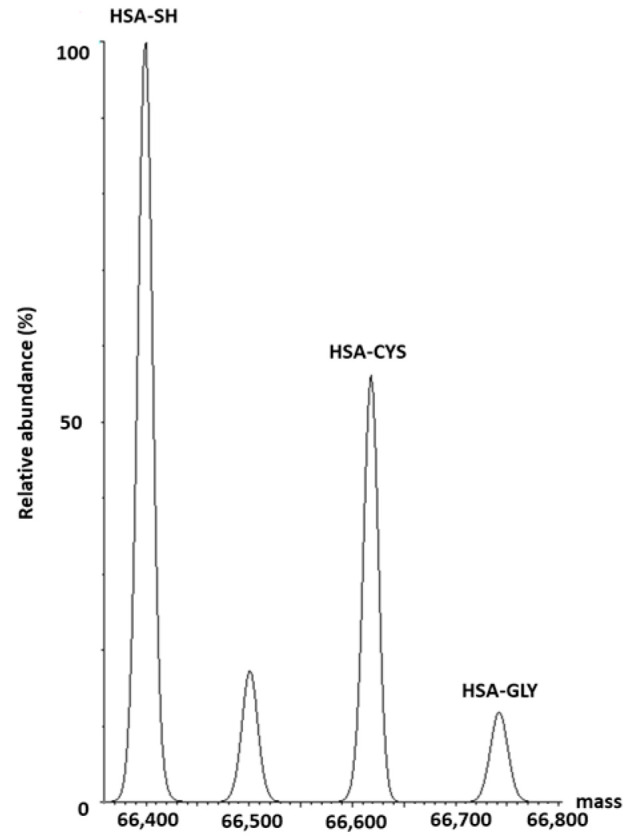
Representative chromatogram of different HSA proteoforms in human HSA.

The different HSA proteoforms, namely, HSA-SH, HSA-Cys, and HSA-Gly, were identified by the MS-based method. A representative chromatogram is shown in Figure 2.

**Figure 3:**
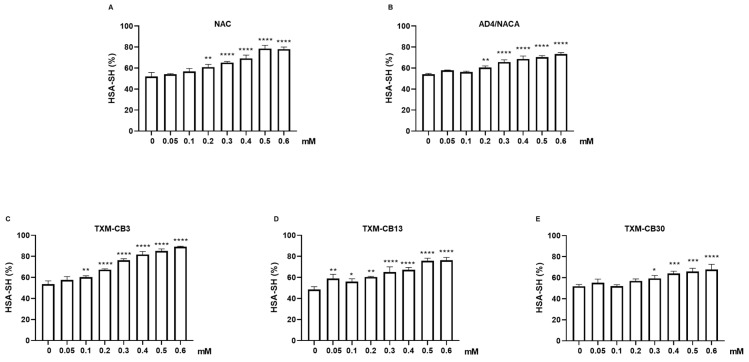
Dose-dependent increase in HSA-SH mediated by N-Acetylcysteine (NAC), N-Acetylcysteine Amide (AD4/NACA), and TXM peptides. HSA solution (200 g/L) was incubated at 37 °C for 1 h in presence or absence of (**A**) NAC, (**B**) AD4/NACA, (**C**) TXM-CB3, (**D**) TXM-CB13, or (**E**) TXM-CB30, as indicated. Levels of HSA-SH were determined by LC–mass spectrometry. *n* = 3. * *p* < 0.05, ** *p* < 0.005, *** *p* < 0.001, **** *p* < 0.0001 vs. 0 by ANOVA, followed by Dunnett’s test for multiple comparison.

HSA treatment with NAC, AD4/NACA, TXM-CB3, TXM-CB13, or TXM-CB30 was performed in a dose-dependent manner in the range of 0.05–0.6 mM and showed an increase in the levels of the reduced proteoform HSA-SH (Figure 3A–E). All the compounds showed a significant increase in HSA-SH levels, even at lower concentration of 0.2–0.3 mM. The maximum increase was observed at 0.6 mM. Among the compounds, TXM-CB3 was found to be most potent, with an increase of 66.96 ± 8.85% in HSA-SH levels at 0.6 mM (Figure 3C).

**Figure 4:**
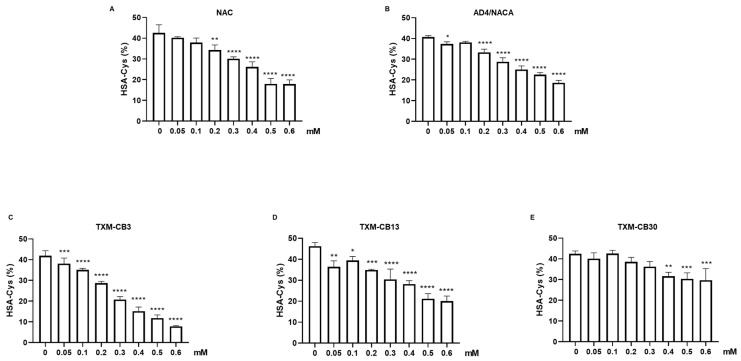
Dose-dependent decrease in HSA-Cys mediated by N-Acetylcysteine (NAC), N-Acetylcysteine Amide (AD4/NACA), and Thioredoxin Mimetic (TXM) peptides. HSA solution (200 g/L) was incubated at 37 °C for 1 h in presence or absence of (**A**) NAC, (**B**) AD4/NACA, (**C**) TXM-CB3, (**D**) TXM-CB13, or (**E**) TXM-CB30 as indicated. Levels of HSA-Cys were determined by LC–mass spectrometry. *n* = 3. * *p* < 0.05, ** *p* < 0.005, *** *p* < 0.001, **** *p* < 0.0001 vs. 0 by ANOVA, followed by Dunnett’s test for multiple comparison.

For all tested compounds, the increase in the HSA-HS levels was correlated with a significant decrease in the HSA-Cys levels, the oxidized proteoform of HSA (Figure 4). TXM-CB3 appeared to be most potent, significantly reducing the HSA-Cys levels already at 0.05 mM (−8.9 ± 7.56%) and peaking at 0.6 mM (−81.24 ± 0.38%). Similarly, all tested compounds displayed their highest effect at 0.6 mM (Figure 4A–E).

### 3.2. Time Dependency of N-Acetylcysteine (NAC), N-Acetylcysteine Amide (AD4/NACA), and Thioredoxin Mimetic (TXM) Peptides TXM-CB3, TXM-CB13, and TXM-CB30 on Levels of Reduced and Oxidized Proteoforms of Albumin

**Figure 5:**
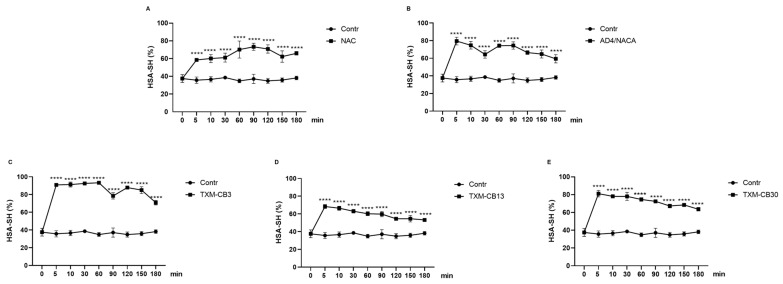
Time dependency of NAC, AD4/NACA, and Thioredoxin Mimetic (TXM) peptides on levels of HSA-SH. HSA solution was incubated at 37 °C in presence or absence of (**A**) NAC, (**B**) AD4/NACA, (**C**) TXM-CB3, (**D**) TXM-CB13, or (**E**) TXM-CB30, at 0.6 mM, at different time points, as indicated. Levels of HSA-HS were determined by LC–mass spectrometry. *n* = 3. **** *p* < 0.0001 vs. 0 by ANOVA, followed by Dunnett’s test for multiple comparison.

The incubation of the HSA solution with 0.6 mM of NAC, AD4/NACA, TXM-CB3, TXM-CB13, or TXM-CB30 resulted in a significant increase in the levels of the reduced proteoform HSA-SH (Figure 5A–E). After 5 min, all the compounds displayed a significant increase. Although the effect was slightly diminished over time, the HSA-SH levels remained significantly high for 180 min compared to the HSA control solution.

**Figure 6:**
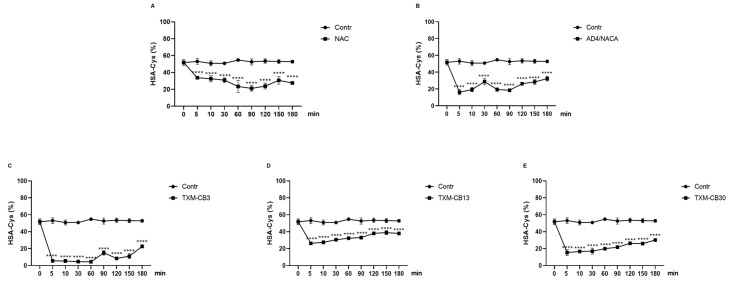
Time dependency of NAC, AD4/NACA, and Thioredoxin Mimetic (TXM) peptides on levels HSA-Cys. HSA was incubated at 37 °C in presence or absence of (**A**) NAC, (**B**) AD4/NACA, (**C**) TXM-CB3, (**D**) TXM-CB13, or (**E**) TXM-CB30, at 0.6 mM, at different time points, as indicated. Levels of HSA-Cys were determined by LC–mass spectrometry. *n* = 3. **** *p* < 0.0001 vs. 0 by ANOVA, followed by Dunnett’s test for multiple comparison.

After 5 min of incubation, all the compounds showed a significant reduction in the HSA-Cys levels, as indicated in Figure 6A–E. The figure demonstrates that the HSA-Cys level was inversely related to the HSA-SH level.

### 3.3. N-Acetylcysteine (NAC), N-Acetylcysteine Amide (AD4/NACA), and Thioredoxin Mimetic Peptides TXM-CB3, TXM-CB13, and TXM-CB30 Increase the Level of Free Sulfhydryl Groups

**Figure 7:**
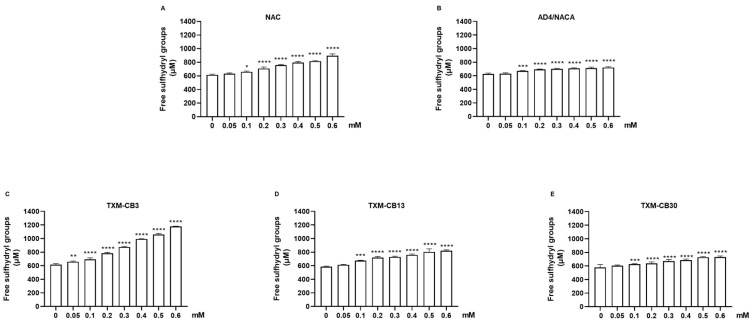
Increase in level of free sulfhydryl groups by NAC, AD4/NACA, and Thioredoxin Mimetic (TXM) peptides. HSA was incubated at 37 °C for 1 h in presence of (**A**) NAC, (**B**) AD4/NACA, (**C**) TXM-CB3, (**D**) TXM-CB13, or (**E**) TXM-CB30 as indicated. Levels of free sulfhydryl groups were detected using Ellman’s reagent. *n* = 3. * *p* < 0.05; ** *p* < 0.01; *** *p* < 0.001; **** *p* < 0.0001 vs. 0 by ANOVA, followed by Dunnett’s test for multiple comparison.

The HSA solution was treated with NAC, AD4/NACA, TXM-CB3, TXM-CB13, or TXM-CB30. The level of free sulfhydryl groups was detected and analyzed as shown in Figure 7. An increase in the level of free sulfhydryl groups was observed at low concentrations ranging from 0.1 to 0.2 mM, except for TXM-CB3, which showed a significant increase at concentrations as low as 0.05 mM (Figure 7C). All compounds showed a dose-dependent effect, with TXM-CB3 being the most potent (Figure 7A,C,D). AD4/NACA and TXM-CB30 demonstrated a significant maximal effect at 0.1 mM (Figure 7B,E).

**Figure 8:**
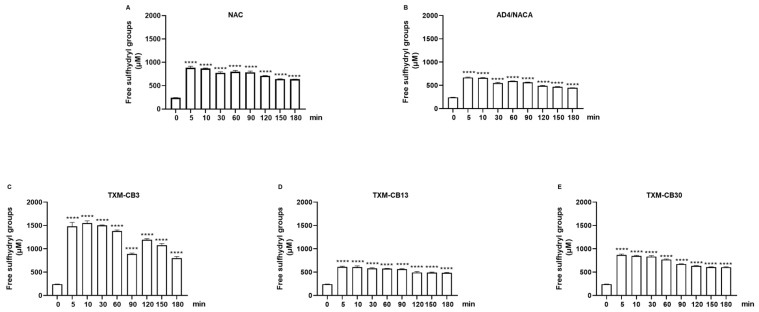
Effect of NAC, AD4/NACA, and Thioredoxin Mimetic (TXM) peptides on free sulfhydryl groups in HSA. HSA solution was incubated with the compounds at 37 °C at different time points in the presence of 0.6 mM (**A**) NAC, (**B**) AD4/NACA, (**C**) TXM-CB3, (**D**) TXM-CB13, or (**E**) TXM-CB30. The level of free sulfhydryl groups was detected using Ellman’s reagent. *n* = 3. **** *p* < 0.0001 vs. 0 by ANOVA, followed by Dunnett’s test for multiple comparison.

A rapid and significant increase in the number of free sulfhydryl groups in HSA was observed in the presence of each of the compounds and was detected within 5 min of incubation (Figure 8A–E). This effect remained significantly high, also after 180 min of incubation, followed by a gradual decline afterwards. Among all the compounds tested, TXM-CB3 was found to be the most potent, showing an increase of approximately 530% in HSA-SH levels after 10 min of incubation (Figure 8C).

### 3.4. Antioxidant Activity of N-Acetylcysteine (NAC), N-Acetylcysteine Amide (AD4/NACA), and Thioredoxin Mimetic (TXM) Peptides TXM-CB3, TXM-CB13, and TXM-CB30

**Figure 9:**
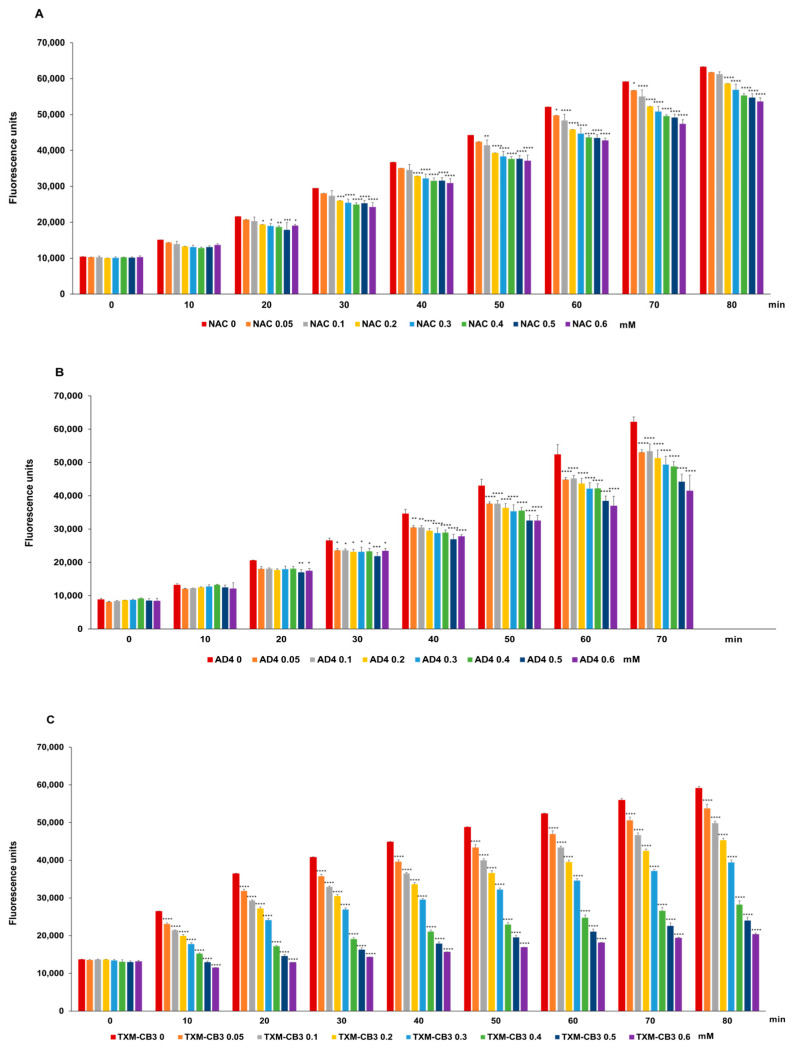

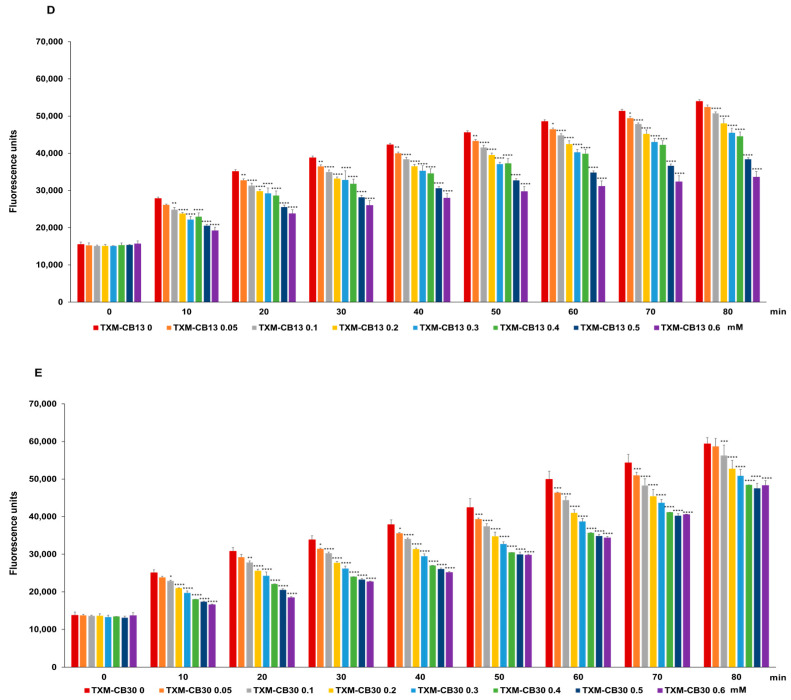
Antioxidant activity of the compounds. HSA solution was preincubated with increasing concentrations (0–0.6 mM) of (**A**) NAC, (**B**) AD4/NACA, (**C**) TXM-CB3, (**D**) TXM-CB13, or (**E**) TXM-CB30 for 1 h at 37 °C. Antioxidant activity was detected via DCF fluorescence. Data are expressed as fluorescence units of DCF. *n* = 3. * *p* < 0.05; ** *p* < 0.01; *** *p* < 0.001; **** *p* < 0.0001 vs. 0 by 2-way ANOVA, followed by Dunnett’s test for multiple comparison.

The incubation of HSA with NAC, AD4/NACA, TXM-CB3, TXM-CB13, or TXM-CB30 revealed antioxidant activity (Figure 9). In a dose-dependent manner, all the compounds exhibited a reduction in radical generation induced by AAPH, which persisted for up to 80 min and aligned with the peak of radical generation. TXM-CB3 emerged as the most powerful compound, significantly decreasing the radical generation at all tested concentrations (Figure 9C). A slight increase in radical generation (55.1 ± 2.0% at 80 min) was observed at the highest concentration (0.6 mM) compared to the control, demonstrating 338.8 ± 5.5% radical formation being reached.

**Figure 10:**
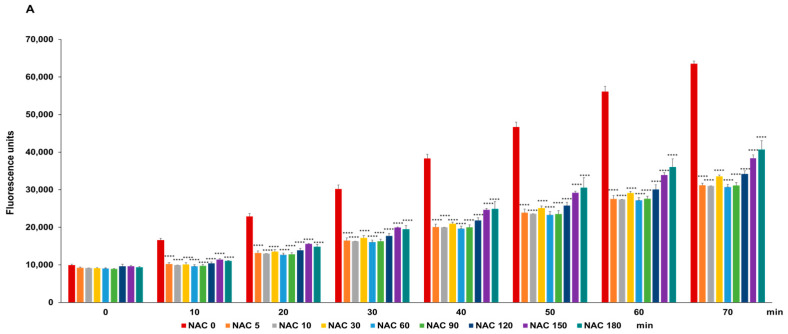

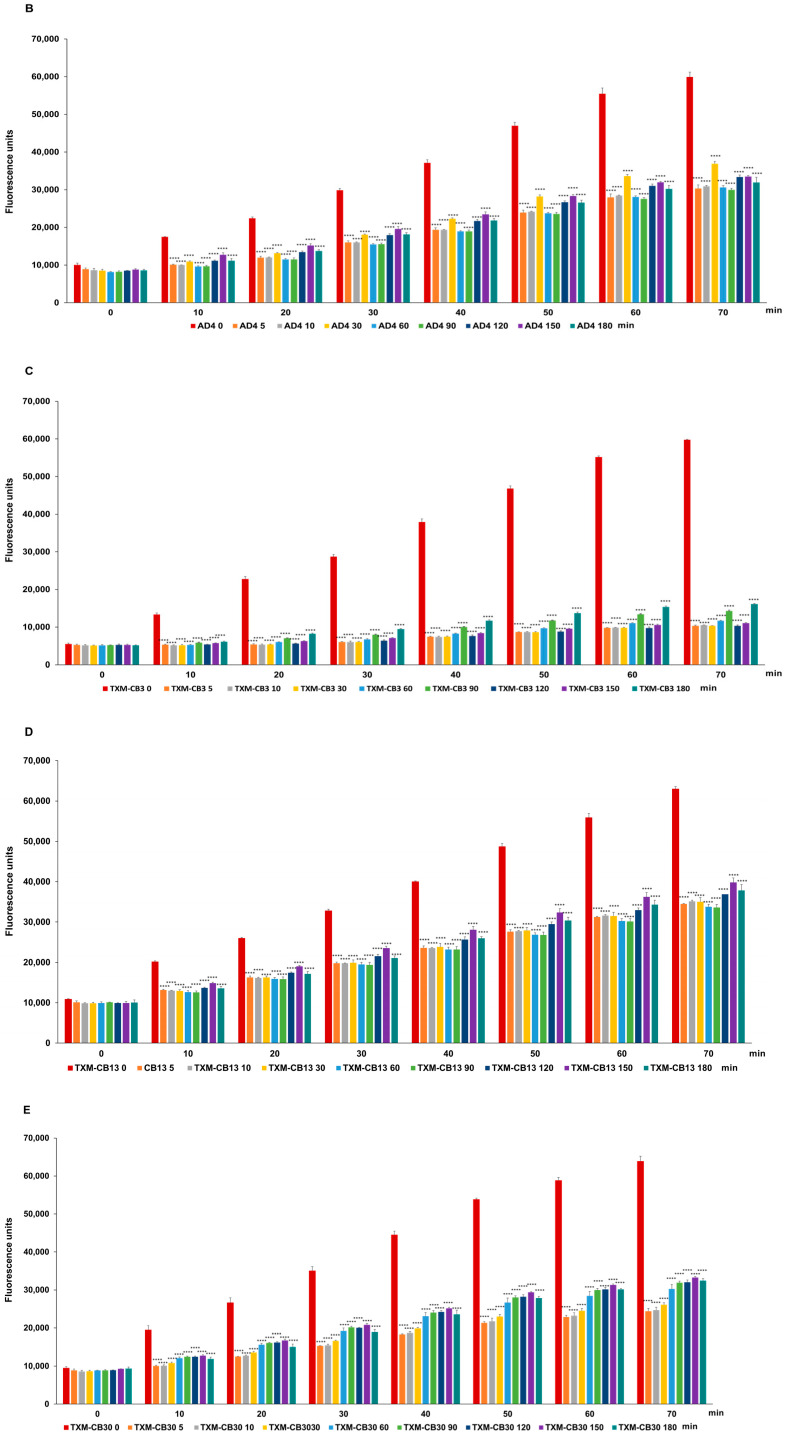
Protection of HSA by the antioxidant activity of the compounds. HSA solution was preincubated at 37 °C for different times with (**A**) NAC, (**B**) AD4/NECA, (**C**) TXM-CB3, (**D**) TXM-CB13, or (**E**) TXM-CB30, all at 0.6 mM. Antioxidant activity was detected via DCF fluorescence. Data are expressed as fluorescence units of DCF. *n* = 3. **** *p* < 0.0001, vs. 0 by 2-way ANOVA, followed by Dunnett’s test for multiple comparison.

The protection of HSA by the redox compounds was performed by incubation of the HSA solution with the compounds at 0.6 mM at different time interval ranging from 0 to 180 min. Subsequently, the radical generator AAPH was added and the DCF fluorescence served as an indicator of radical production, which was monitored for a duration of 70 min (Figure 10). The antioxidant activity of all the compounds appeared already after 5 min of incubation and persisted for longer incubation periods, with only slight variations with incubation times of up to 180 min. TXM-CB3 exhibited a significant decrease in radical generation 10 min after the addition of AAPH (−54.2 ± 2.62), reaching −73.0 ± 0.34 after 70 min (Figure 10C).

### 3.5. The Antioxidant Activity of N-Acetylcysteine (NAC), N-Acetylcysteine Amide (AD4/NACA), and Thioredoxin Mimetic (TXM) Peptides TXM-CB3, TXM-CB13, and TXM-CB30 Is Associated with the Levels of the Reduced Form of Albumin

**Figure 11:**
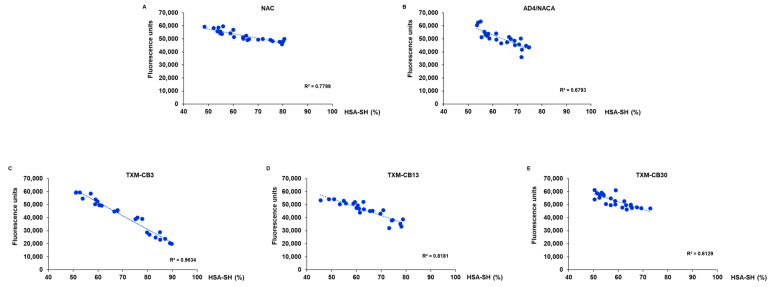
Correlation between antioxidant activity of compounds and HSA-SH levels. HSA solution was preincubated at 37 °C for 1 h with (**A**) NAC, (**B**) AD4/NACA, (**C**) TXM-CB3, (**D**) TXM-CB13, or (**E**) TXM-CB30 in range of 0–0.6 mM. Antioxidant activity was detected via DCF fluorescence. Data are expressed as fluorescence units of DCF. Levels of HSA-SH were measured by LC–mass spectrometry. *n* = 3.

The antioxidant activity of the compounds was measured 70–80 min after the addition of AAPH, at the peak of radical generation. The redox activity was found to be correlated with the levels of HSA-SH, which were detected following incubation of the HSA solution with NAC, AD4/NACA, TXM-CB3, TXM-CB13, or TXM-CB30 (Figure 11).

### 3.6. N-Acetylcysteine (NAC), N-Acetylcysteine Amide (AD4/NACA), and Thioredoxin Mimetic (TXM) Peptides TXM-CB3, TXM-CB13, and TXM-CB30 Reduce HSA-Cys and Increase Free Sulfhydryl Groups in Plasma

**Figure 12:**
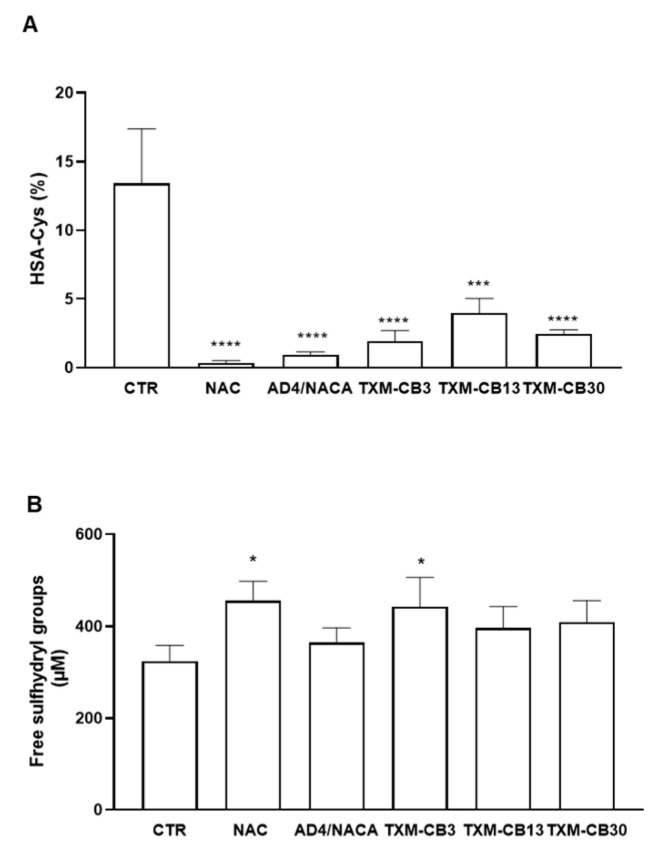
Effect of NAC, AD4/NACA, and Thioredoxin Mimetic (TXM) peptide on levels of HSA-Cys and free sulfhydryl groups in plasma. Plasma obtained from healthy subjects was incubated with the compounds at 37 °C in presence of 0.6 mM NAC, AD4/NACA, TXM-CB3, TXM-CB13, or TXM-CB30. (**A**) Levels of HSA-Cys were determined by LC–mass spectrometry. (**B**) Free sulfhydryl groups were detected using Ellman’s reagent. *n* = 3. * *p* < 0.05, *** *p* < 0.001, **** *p* < 0.0001 vs. CTR by ANOVA, followed by Dunnett’s test for multiple comparison.

Plasma obtained from healthy subjects was incubated with NAC, AD4/NACA, TXM-CB3, TXM-CB13, or TXM-CB30. All compounds significantly reduced the levels of HSA-Cys (Figure 12A). Additionally, a significant increase in the levels of free sulfhydryl groups in plasma was detected for each compound compared with vehicle-treated plasma (456.1 ± 41.9 µM for NAC, *p* < 0.001; 364.4 ± 32.2 µM for AD4, *p* < 0.05; 442.4 ± µM for TXM-CB3, *p* < 0.05; 396.0 ± 47.1 µM for TXM-CB13, *p* < 0.05; 408.9 ± 46.8 µM for TXM-CB30, *p* < 0.01; 323.2 ± 35.2 µM for vehicle-treated plasma, paired Student’s *t*-test. *n* = 3).

The comparison among all compounds evaluated by ANOVA revealed that NAC and TXM-CB3 were the most potent compounds, leading to an increase of 41.3 ± 2.4% and 36.5 ± 5.0 for NAC and TXM-CB3, respectively (Figure 12B).

## 4. Discussion

The redox status of HSA can offer a valuable insight into overall pathophysiological conditions, mainly due to anti-inflammatory and antioxidant properties [68,69,70]. Research has demonstrated that the oxidized form of HSA, apart from being an aggravating factor itself, could also play a role in the development of various diseases [8,69,71]. Based on these findings, regeneration of the reduced proteoform of HSA could become an effective therapeutic strategy for enhancing antioxidant defenses and combating oxidative stress-related conditions. Our recent findings demonstrated that NAC treatment significantly reduced the HSA-Cys levels both in vitro and in vivo, with a concomitant increase in native HSA-SH levels [38,40].

### 4.1. AD4/NACA

Previously, the antioxidant properties of AD4/NACA have been extensively investigated through in vitro and in vivo studies in different cell lines and animal models [46]. Compared to NAC, AD4/NACA showed a higher 2,2-diphenyl-1-picryl-hydrazyl-hydrate (DPPH) radical scavenging capability and reducing power [72]. Additionally, AD4/NACA showed a superior H_2_O_2_ scavenging capacity at the highest tested concentration (500 g/mL), while NAC performed better at lower concentrations (125 µM and 250 µM) [73]. AD4/NACA has been shown to protect red blood cells from thiol depletion induced by tert-Butyl hydroperoxide [39]. Studies in β-thalassemic cells have demonstrated the superior efficacy of AD4/NACA in reducing reactive oxygen species (ROS) generation and elevating glutathione (GSH) levels when compared to NAC [74].

In a rat model of ischemia–reperfusion injury, it was demonstrated that NAC and AD4/NACA had similar protective activity on oxidative damage and erythrocyte deformability [75]. In particular, treatment with AD4/NACA restored 91% of endogenous thiols in erythrocytes, while NAC reached only 15%. In contrast to NAC, AD4/NACA protected tert-Butyl hydroperoxide-treated erythrocytes from hemoglobin oxidation [75]. Based on cell-free assays, the thiol–disulfide exchange between AD4/NACA and oxidized glutathione has been proposed as a mechanism underlying the antioxidant effect of AD4/NACA [47].

### 4.2. TXM Peptides

Overexpression of Trx1 has been demonstrated to effectively protect against oxidative stress-induced cellular damage in several animal models of oxidative and inflammatory disorders. Also, the administration of recombinant Trx1 protein was effective in mitigating inflammatory conditions such as acute lung injury, suggesting a potential clinical application of the recombinant Trx1 [46,76].

Owing to its structural resemblance to the Trx catalytic motif, specifically the sequence Cys-Pro-Cys, which represents a truncated form of the -Cys-Pro-Gly-Cys- catalytic motif found in Trx1, it has been postulated that TXM-CB3, similar to other TXM peptides, can mimic the dithiol–disulfide exchange reaction of Trx. This process involves the participation of two redox-active Cys residues in maintaining the redox state of the cell [64].

The antioxidant efficacy of TXM-CB3 has been substantiated in vivo in an allergic airway model, where it effectively up-regulated GSH levels and inhibited NF-κB and p38 MAPK activity [77].

Additionally, intraperitoneal injections of TXM-CB3 into ApoE2.Ki mice that were fed a high-fat diet reduced the plasma levels of antibodies against oxidized low-density lipoproteins and atherosclerotic lesions, as well as the number of pro-inflammatory macrophages in the aortic sinus [52]. TXM-CB3 has been shown to improve endothelial cell function in diabetes [78] and to improve mouse cardiac function, reducing the size of cardiac infarct and fibrosis and decreasing the expression of cardiac inflammatory markers [58].

Furthermore, as a result of the synergistic effect with NADPH activity, TXM-CB3 is also capable of denitrosylating small-molecular-weight S-nitrosothiol compounds, such as S-nitrosoglutathione [79]. As the denitrosylation activity of the Trx/TrxR system can modulate both NO-based signaling and nitrosative stress [80,81], the denitrosylation activity of this compound suggests an additional potential therapeutic application for TXM-CB3 in diseases associated with nitrosative stress.

In the present study, we demonstrate that TXM-CB3, TXM-CB13, and TXM-CB30 effectively restore HSA-SH and correspondingly reduce HSA-Cys levels. Remarkably, TXM-CB3 exhibited the highest efficacy among all the tested compounds, potentially attributed to the Pro residue, a characteristic hallmark of this peptide. Different structural features of the peptides may contribute to reduced susceptibility to proteolysis such as Pro residue in TXM-CB3, or the D-isomer configuration in TXM-CB3. Additional studies are required to understand the contribution of the peptide amino acid composition to biological efficacy.

## 5. Conclusions

In this study, we propose a novel therapeutic role for AD4/NACA and TXM peptides in protection against oxidative stress-related disorders through increasing the HSA-SH levels, which inversely corresponds to reducing the HSA-Cys levels. These compounds facilitate regeneration of the reduced proteoform within 5 min of the incubation period and demonstrate a higher efficacy compared to NAC. Additionally, each of these compounds induce an elevation in the level of free sulfhydryl groups in HSA, thereby increasing its antioxidant activity, as evaluated by the DCF formation.

Of interest, the antioxidant properties of AD4/NACA, as well as those of TXM peptides, have also been confirmed in biological fluids. Indeed, all these compounds significantly reduce the levels of HSA-Cys and increase the levels of free sulfhydryl groups in plasma.

Although further research is needed, we propose a potentially promising therapeutic application for these small molecular thiol reagents in the treatment of oxidative stress-associated conditions.

## Figures and Tables

**Table 1:** Gradient used for LC-MS analysis.

Time (min)	Flow Rate (µL/min)	%A	%B
Initial	5.00	70.0	30.0
0.60	5.00	70.0	30.0
1.38	5.00	40.0	60.0
8.16	5.00	5.0	95.0
10.20	5.00	5.0	95.0
10.26	5.00	70.0	30.0
16.00	5.00	70.0	30.0

## Data Availability

Data collected in the study will be made available using the data repository Zenodo (https://zenodo.org, accessed on 12 March 2024) with restricted access upon request to direzione.scientifica@ccfm.it. Any remaining information can be obtained from the corresponding author upon reasonable request.
